# Stress hormone rapidly tunes synaptic NMDA receptor through membrane dynamics and mineralocorticoid signalling

**DOI:** 10.1038/s41598-017-08695-3

**Published:** 2017-08-14

**Authors:** Lenka Mikasova, Hui Xiong, Amber Kerkhofs, Delphine Bouchet, Harm J. Krugers, Laurent Groc

**Affiliations:** 1 University de Bordeaux, Interdisciplinary Institute for Neuroscience, UMR 5297, F-33000 Bordeaux, France; 2 University de Bordeaux, Interdisciplinary Institute for Neuroscience, CNRS, IINS UMR 5297 Bordeaux, France; 3 Swammerdam Institute for Life Sciences, University of Amsterdam, Amsterdam, The Netherlands

## Abstract

Stress hormones, such as corticosteroids, modulate the transmission of hippocampal glutamatergic synapses and NMDA receptor (NMDAR)-dependent synaptic plasticity, favouring salient behavioural responses to the environment. The corticosterone-induced synaptic adaptations partly rely on changes in NMDAR signalling, although the cellular pathway underlying this effect remains elusive. Here, we demonstrate, using single molecule imaging and electrophysiological approaches in hippocampal neurons, that corticosterone specifically controls GluN2B-NMDAR surface dynamics and synaptic content through mineralocorticoid signalling. Strikingly, extracellular corticosterone was sufficient to increase the trapping of GluN2B-NMDAR within synapses. Functionally, corticosterone-induced potentiation of AMPA receptor content in synapses required the changes in NMDAR surface dynamics. These high-resolution imaging data unveiled that, in hippocampal networks, corticosterone is a natural, potent, fast and specific regulator of GluN2B-NMDAR membrane trafficking, tuning NMDAR-dependent synaptic adaptations.

## Introduction

Exposure to stress affects cognitive processes partly through the release of stress hormones such as corticosterone (cortisol in humans) and (nor)epinephrine^1, 2^. The stress hormone corticosterone first activates the high-affinity mineralocorticoid receptor (MR) and then, with increasing concentrations as seen during stress the lower-affinity glucocorticoid receptor (GR). These intracellular receptors act as transcription factors and regulate gene expression. In addition, recent evidence demonstrated the presence of membrane MR-like signalling that can mediate fast non-genomic actions of corticosterone^3^. In the brain, corticosteroids are well-known regulators of hippocampal excitatory synaptic transmission and plasticity^4^. Within minutes after exposure, corticosterone reversibly increases the presynaptic glutamate release through membrane MR^5^ and facilitates the long-term potentiation of glutamatergic synapses (LTP) in hippocampal neurons^6^. Within hours, corticosterone increases selectively AMPA receptor (AMPAR) mediated postsynaptic transmission through intracellular GR^7^, impairing LTP^8^. Remarkably, these time-dependent effects of corticosterone on synaptic adaptation require surface mobilization of AMPARs through an alteration of their membrane delivery and surface diffusion^4^.

The plasticity of glutamatergic synapses often requires the activation of the calcium permeable postsynaptic NMDA receptors (NMDAR). In addition to the binding of glutamate and co-agonists (D-serine and glycine), this receptor requires membrane depolarization to remove the voltage-dependent magnesium block and permit ion fluxes. The combined requirement for glutamate and postsynaptic depolarization enables NMDAR to detect coincident pre- and postsynaptic activities, a paradigm of Hebbian synaptic plasticity such as LTP^9^. The NMDAR are heterotetramers comprising various combinations of GluN1, GluN2A-D, and GluN3 subunits, which confer specific biophysical and pharmacological properties to the receptor^10^. In hippocampal glutamatergic synapses, the presence of GluN2A- and GluN2B-NMDAR differentially influences synaptic plasticity^11^, although the precise role and molecular mechanism underlying this process remain highly debated. The ratio between GluN2A- and GluN2B-NMDAR is not uniform among synapses, across brain structures, and throughout brain development^12^, likely reflecting different metaplastic states and history between synapses. In addition to these long-term changes, modifications of the 2A/2B synaptic ratio have been reported to occur shortly (few minutes) after the induction of synaptic plasticity in young hippocampal neurons^13, 14^. These fast changes likely involve rapid alterations of receptor trafficking, through exocytosis/endocytois and/or surface diffusion. At the plasma membrane level, NMDAR diffuse in a GluN2 subunit-dependent manner and explore rather large area around synaptic area^15–17^, supporting the current view that the surface dynamics of GluN2-NMDAR controls the plastic range of glutamatergic synapses.

It has previously been demonstrated that corticosteroids regulate the GluN2A/2B synaptic ratio over time through various signalling cascades^18–21^, constituting the cellular ground for stress hormone regulation of synaptic plasticity. Here, we investigated the cellular pathway by which such regulatory events occur. We directly tested whether corticosterone alters the distribution and dynamics of NMDAR subtypes at the plasma membrane of hippocampal neurons, by focusing on the GluN2A and GluN2B subunits following corticosterone exposure using single nanoparticle tracking and electrophysiological approaches.

## Results

### Corticosterone acutely increases GluN2B-NMDAR, but not GluN2A-NMDAR, surface and synaptic content

**Figure 1:**
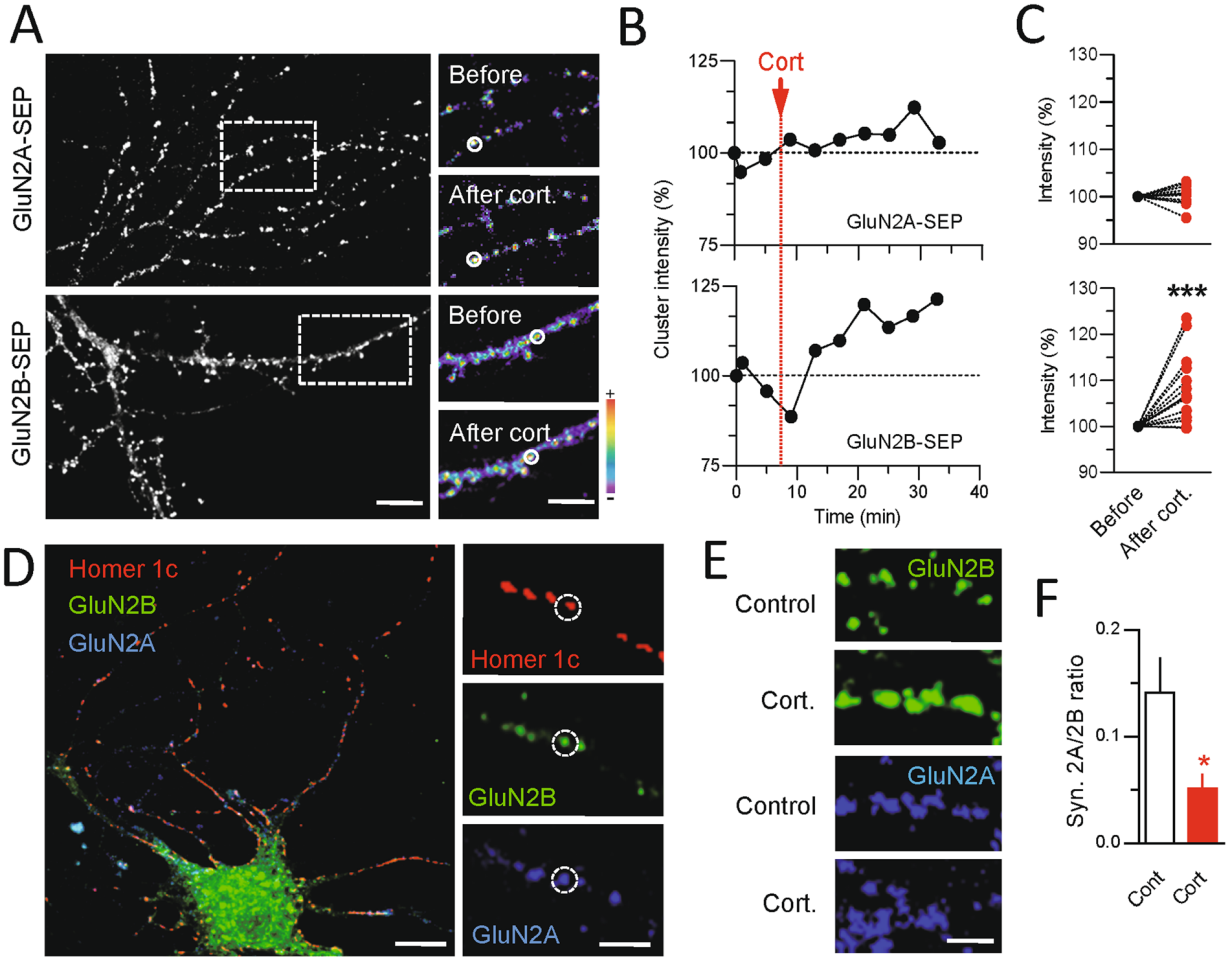
Corticosterone alters surface GluN2B-NMDAR clustering. (**A**) Dendritic fragments of GluN2A- and GluN2B-SEP expressing neurons before and after exposure to corticosterone (100 nM). Scale bar = 5 µm, scale bar inset = 1 µm. (**B**) Example of fluorescence intensity of GluN2A- (n = 12 dendritic fields, N = 5 neurons) and GluN2B-SEP (n = 15 dendritic fields, N = 5 neurons) clusters over time. (**C**) Comparison of GluN2A- and GluN2B-SEP cluster fluorescence intensity before and after exposure to corticosterone. ***p < 0.001, paired Student t-test. (**D**) Live immunostaining of GluN2A and GluN2B subunits in Homer 1c-DsRed expressing neurons. Scale bar = 10 µm, scale bar inset = 5 µm. (**E**) Immunostaining of surface GluN2A and GluN2B subunits before and after exposure to corticosterone. Scale bar = 5 µm. (**F**) Comparison of the fluorescence intensity of GluN2A (n = 12 dendritic fields, N = 5 neurons) and GluN2B (n = 11 dendritic fields, N = 5 neurons) subunit clusters (expressed as ratio) -SEP before and after exposure to corticosterone (50 nM, 20 min). *p < 0.05, Student t-test.

To investigate the acute effect of corticosterone on the distribution of NMDAR subtypes embedded into the plasma membrane of live hippocampal neurons, we over-expressed the GluN2A or GluN2B subunit fused to a Super Ecliptic pHluorin (SEP) at its extracellular N-terminus (GluN2A and GluN2B-SEP) to image preferentially (not exclusively, see ref. 22) the surface receptor pool^23^. The fluorescence intensity of clustered GluN2A- or GluN2B-NMDAR, which has been shown to co-localize with synaptic markers^24^, was measured over time before and after exposure to 50 nM corticosterone (Fig. 1A). GluN2A-NMDAR clusters were stable over the time within the 30 min period following corticosterone exposure (Fig. 1A–C). In contrast, the fluorescence of GluN2B-NMDAR clusters rapidly and significantly increased already 5 min after corticosterone exposure (Fig. 1A–C). This increase was stable over 30 min as it remained to its highest value during this period (Fig. 1B). Thus, corticosterone acutely increases the clustering of surface GluN2B-NMDAR. We directly measured the surface level of GluN2A- and GluN2B-NMDAR content in glutamate synapse by transfecting neurons with GluN2A and GluN2B subunits containing different extracellular tags and the postsynaptic protein Homer 1c-DsRed (Fig. 1D). After live immunocytochemical staining of the surface GluN2 subunits, corticosterone exposure increased the staining of synaptic GluN2B-NMDAR, significantly decreasing the GluN2A/2B ratio within glutamate synapses (Fig. 1E,F). Collectively, these data indicate that a single acute corticosterone exposure rapidly alters the GluN2A/2B synaptic ratio through a specific alteration of the clustering of GluN2B-NMDAR.

### Corticosterone increases NMDAR-mediated miniature EPSC and sensitivity to a GluN2B antagonist

**Figure 2:**
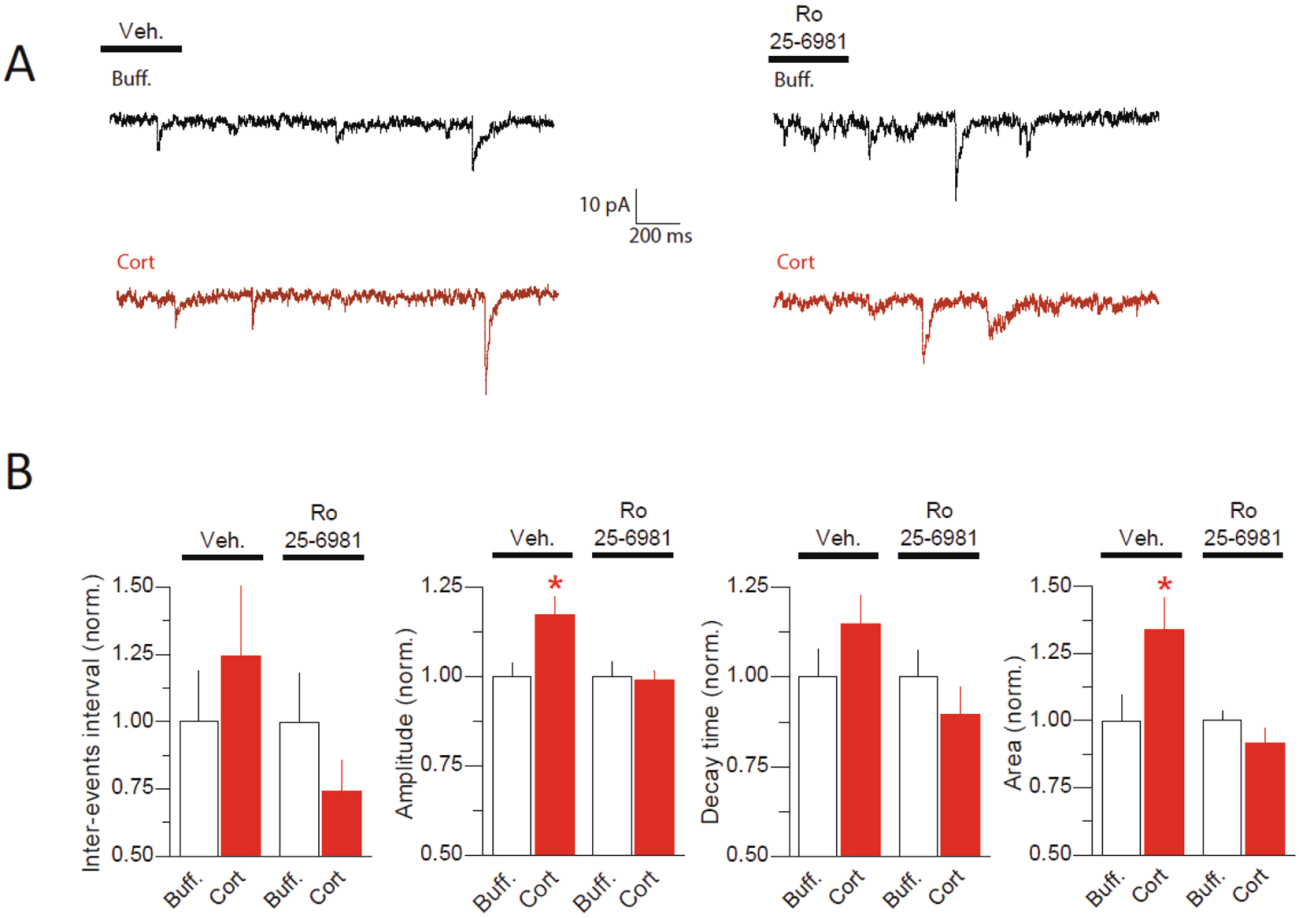
Corticosterone increases amplitude and charged area of NMDAR-mediated mEPSCs. (**A**) Representative traces of NMDAR mEPSCs after vehicle (<0.1% ethanol) and corticosterone (100 nM), in the presence or absence of Ro25–6981. Scale bar: horizontal 200 ms, vertical 10 pA. (**B**) Comparison of the inter-event interval, amplitude, decay time and area of NMDAR mEPSCs. mEPSCs were recorded in buffer without (vehicle, n = 14 cells) and with corticosterone (n = 19 cells, red bars). In a separate series of experiments, NMDAR mEPSCs were recorded in the presence of Ro 25–6981 (3 μM, n = 9 cells) or Ro 25–6981 (3 μM) plus corticosterone (100 nM) (n = 7 neurons). *p < 0.05, unpaired Student *t*-test.

To examine the functional effect of corticosterone on the synaptic NMDAR-mediated current, corticosterone (100 nM) was applied to hippocampal cultured neurons and NMDAR-mediated miniature EPSCs (NMDAR mEPSCs) were recorded in neurons (Fig. 2A) (see Methods for details). After 20 min, the frequency of NMDAR mEPSC was unaltered (Fig. 2B). However, corticosterone significantly increased the peak amplitude and charge (area under the curve) of NMDAR mEPSCs when compared to vehicle-treated cells (Fig. 2B). The decay times of NMDAR mEPSCs tended to increase, although not significantly, after corticosterone exposure (Fig. 2B). Thus, corticosterone acutely increased the NMDAR-mediated current in spontaneously active glutamate synapses, with a putative recruitment of NMDAR with slower decay kinetics. To specifically test whether GluN2B-NMDAR are recruited following corticosterone exposure, Ro 25–6981 (potent and selective activity-dependent blocker of GluN2B-NMDAR) was bath-applied and the corticosterone-induced changes in NMDAR mEPSCs were measured (Fig. S1). In the presence of Ro 25–6981 corticosterone administration did not alter NMDAR-mEPSCs (Fig. 2G–J), indicating that corticosterone rapidly promotes GluN2B-NMDAR mediated current. Noteworthy, the access resistance was not different between vehicle (23.6 ± 1.7 MΩ) and corticosterone (24.1 ± 2.1 MΩ) treated cells or between Ro-25 (21.3 ± 2.3 MΩ) and Ro-25+corticosterone (20.7 ± 1.6 MΩ) treated cells.

### Corticosterone rapidly alters the surface dynamics of NMDAR

**Figure 3:**
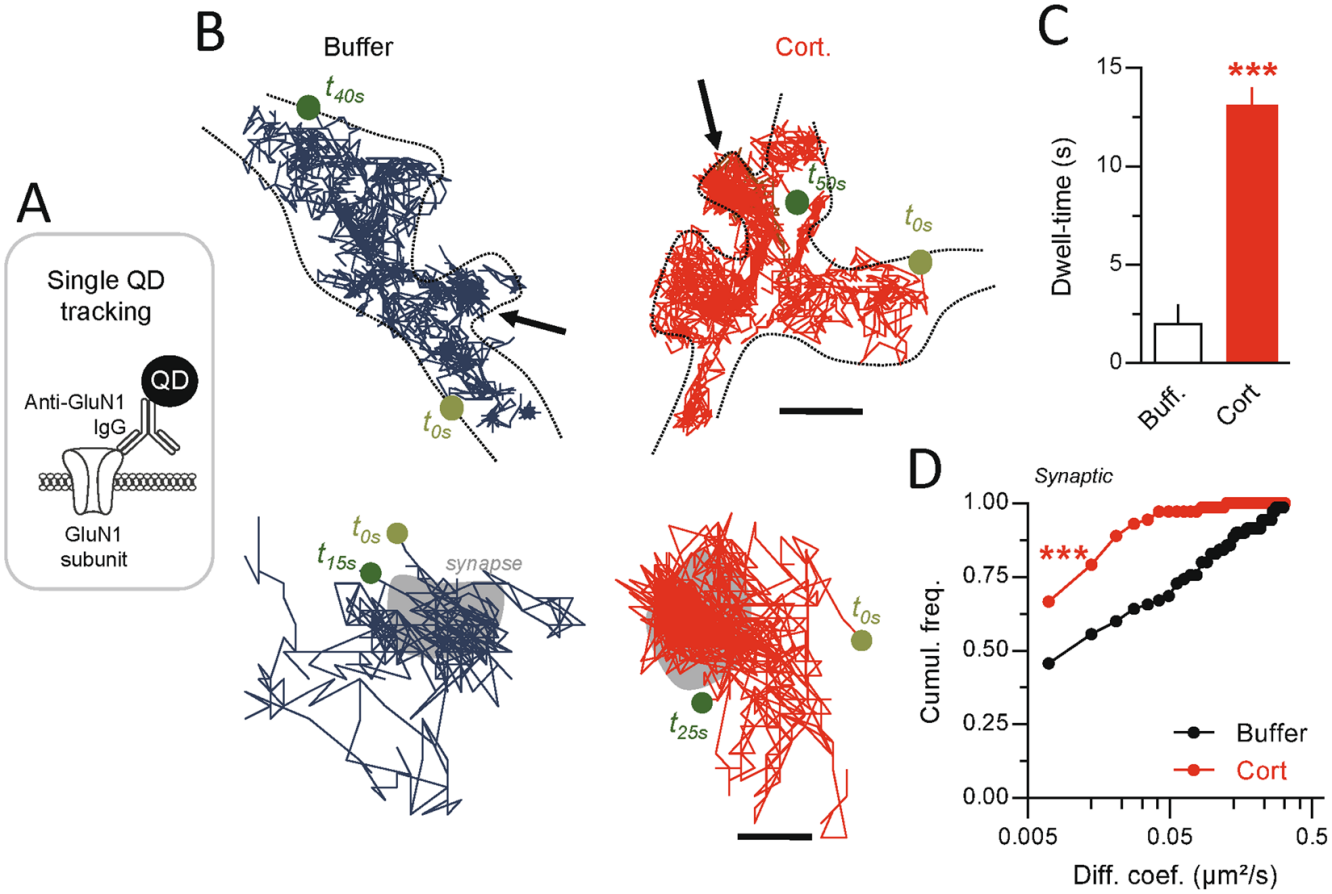
Corticosterone decreases the surface dynamics of GluN1-NMDAR in hippocampal neurons exposed to corticosterone. (**A**) Schematic representation of antibody against GluN1 subunit and single QD complex used to label and track surface NMDAR. (**B**) Representative trajectories of single GluN1-NMDAR in control (buffer, blue) and corticosterone (100 nM, 20 min; red). Note that the traces represent different receptors. The black arrows point toward spines in which glutamatergic synapses were identified. Lower panels, enlarged trajectories located within the postsynaptic densities (gray areas). Starting and ending time of the single trajectories are indicated as for instance time 0 (t_0s_). (**C**) Comparison of the synaptic dwell-time (expressed in seconds) of surface GluN1-NMDAR in buffer (n = 55 trajectories) or corticosterone (n = 62 trajectories) condition. ***p < 0.001, Student t-test. (**D**) Comparison of the cumulative distributions of GluN1-NMDAR instantaneous diffusion coefficients in buffer (n = 172 trajectories) and corticosterone (n = 190 trajectories) conditions. ***p < 0.001, Kolmogorov-Smirnov test.

One possible mechanism underlying the corticosterone-induced increase in GluN2B-NMDAR current is an alteration of the receptor trafficking. Indeed, corticosterone acutely modulates the surface dynamics of another glutamate receptor, i.e. the AMPA type^25, 26^. Furthermore, the synaptic pool of NMDAR depends on the receptor trafficking^27^. Once at the plasma membrane, NMDAR diffuse within the plasma membrane, a process that continuously provides receptor to synaptic areas^28, 29^. To test whether corticosterone acutely modulates surface NMDAR in live hippocampal neurons, we first tracked endogenous surface GluN1 subunit (obligatory subunit of NMDAR) using a single nanoparticle detection approach^30, 31^ (Fig. 3A). In the buffer (PBS) condition, most GluN1-NMDAR exhibited a lower trafficking within glutamate synapse areas when compared to the extrasynaptic compartment (Fig. 3B), likely due to the anchoring by synaptic scaffold proteins^32, 33^. Strikingly, corticosterone (50 nM, 20 min) reduces the surface dynamics of GluN1-NMDAR in the synaptic (Fig. 3B). In presence of corticosterone, GluN1-NMDAR synaptic dwell-time (time spent within the postsynaptic area) was significantly increased and the diffusion coefficients of synaptic GluN1-NMDAR consistently decreased (Fig. 3C,D). The diffusion down-regulation was mostly explained by the up-shift of the immobile fraction, indicating a higher fraction of immobile NMDAR. Together, these data indicate thus that corticosterone rapidly reduces the surface dynamics of NMDAR within glutamate synapses, favoring their active retention and anchoring.

### Corticosterone specifically alters GluN2B-NMDAR surface dynamics and synaptic stabilization through a MR-dependent mechanism

**Figure 4:**
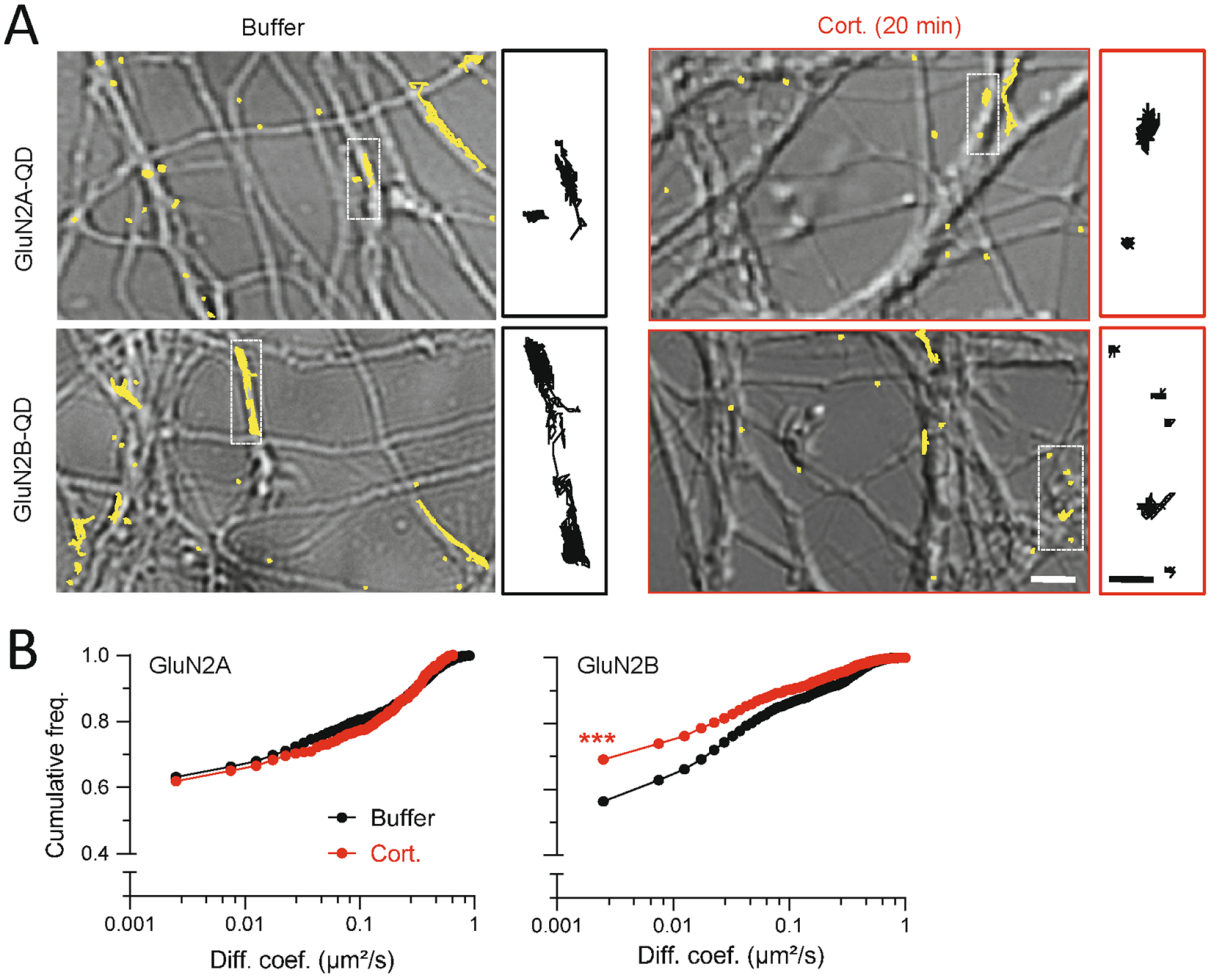
Corticosterone specifically decreases the surface dynamics of GluN2B-NMDAR in hippocampal neurons exposed to corticosterone. (**A**) Representative trajectories of single GluN2A- and GluN2B-NMDAR in hippocampal neurons (DIC images) exposed to either buffer or corticosterone (100 nM, 20 min). Scale bar, 5 µm; scale bar inset, 1 µm. (**B**) Comparison of the cumulative distributions of GluN2A- (buffer, n = 112 trajectories; corticosterone, n = 143 trajectories) and GluN2B-NMDAR (buffer, n = 189 trajectories; corticosterone, n = 191 trajectories) instantaneous diffusion coefficients in buffer and corticosterone conditions. ***p < 0.001, Kolmogorov-Smirnov test.

As shown above, corticosterone alters the surface distribution of GluN2B-NMDAR and the surface dynamics of GluN1-NMDAR (Figs 1 and 3). One may suggest that the corticosterone-induced GluN2B-NMDAR redistribution relies on a change in GluN2B-NMDAR surface dynamics. To test this possibility, we tracked single GluN2B-NMDAR at the surface of live hippocampal neurons. Consistently, corticosterone exposure (50 nM, 20 min) significantly decreased GluN2B-NMDAR surface diffusion whereas no change was observed for GluN2A-NMDAR diffusion (Fig. 4A,B).

**Figure 5:**
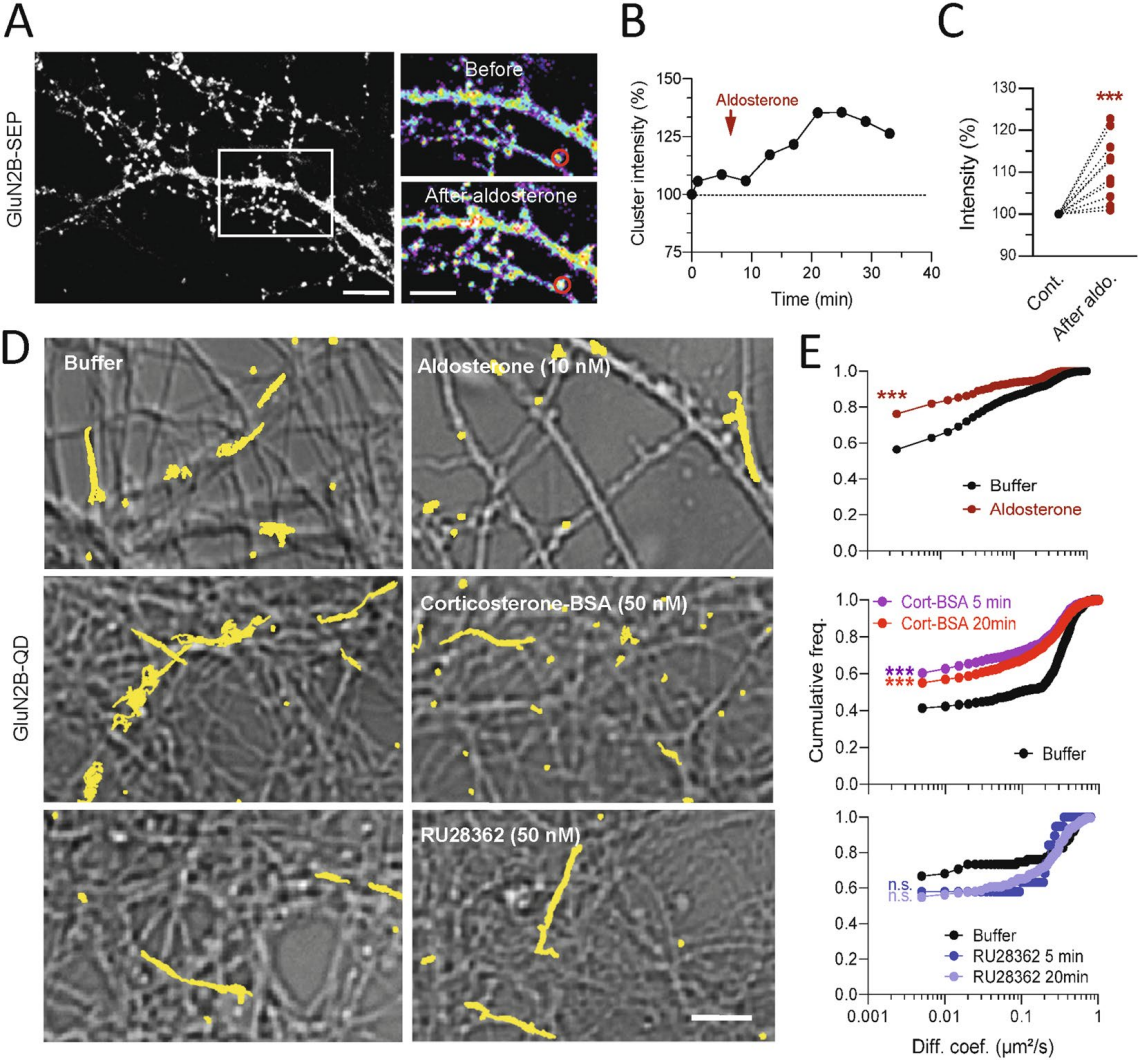
Aldosterone, a MR activator, alters the surface distribution and dynamics of GluN2B-NMDAR. (**A**) Dendritic fragments of GluN2B-SEP expressing neurons before and after exposure to aldosterone (10 nM). Scale bar = 5 µm, scale bar inset = 1 µm. (**B**) Example of fluorescence intensity of GluN2B-SEP clusters over time, before and after aldosterone application. (**C**) Comparison of GluN2B-SEP cluster fluorescence intensity before and after exposure to aldosterone (10 nM, 25 min exposure; n = 10 dendritic fields, N = 5 neurons). ***p < 0.001, paired Student t-test. (**D**) Representative trajectories of single GluN2B-NMDAR in hippocampal neurons (DIC images) exposed to either buffer solution, aldosterone (10 nM, 20 min), corticosterone BSA (50 nM, 20 min), or RU28392 (GR agonist, 50 nM, 20 min). Scale bar, 5 µm. (**E**) Comparison of the cumulative distributions of GluN2B-NMDAR instantaneous diffusion coefficients between aldosterone (buffer, n = 214 trajectories; aldosterone, n = 225 trajectories), corticosterone BSA (buffer, n = 539 trajectories; 5min corticosterone BSA, n = 855 trajectories; 20min corticosterone BSA, n = 864 trajectories), or RU28392 (buffer, n = 229 trajectories; 5min RU28392, n = 175 trajectories; 20min RU28392, n = 119 trajectories). ***p < 0.001, Kolmogorov-Smirnov test.

To dissect the upstream event that lead to this rapid redistribution of surface GluN2B-NMDAR after corticosterone exposure, we investigated whether a MR agonist (aldosterone, 10 nM, 20 min) was able to produce similar effects. Indeed, it was previously demonstrated in hippocampal neurons that corticosterone rapidly alters the surface trafficking of AMPA receptor through a MR-like pathway^25, 26^. Aldosterone exposure produced a rapid and stable increase in the fluorescence of GluN2B-NMDAR surface clusters (Fig. 5A–C), similar to the time-course and magnitude of the effect observed after corticosterone (Fig. 1A). This suggests that corticosterone effect on GluN2B-NMDAR surface distribution is mediated by a MR-like signaling pathway. Surface GluN2B-NMDAR were then tracked using single QD imaging in absence or presence of aldosterone. A rapid and significant reduction of GluN2B-NMDAR surface diffusion, similar to the effect produced by corticosterone, was observed with aldosterone (Fig. 5D,E). To test whether these effects were specific to the MR signaling, neurons were exposed to a GR agonist, RU28362 (50 nM). In contrast to aldosterone, RU28362 did not alter GluN2B-NMDAR surface dynamics both 5 and 20 min after GR activation (Fig. 5D,E). Because corticosterone can rapidly cross the plasma membrane and act intracellularly on signaling cascade, we exposed neurons to corticosterone coupled to bovine serum albumin (BSA) (50 nM, 20 min), which is a membrane non-permeate active analog of corticosterone. Strikingly, corticosterone-BSA rapidly decreased the surface diffusion of GluN2B-NMDAR with a significant and maximal effect already observed 5 min after exposure (Fig. 5D,E). Collectively, these data demonstrate that the GluN2B-NMDAR surface dynamics is rapidly and strongly affected by corticosterone through a membrane MR-like signaling pathway.

**Figure 6:**
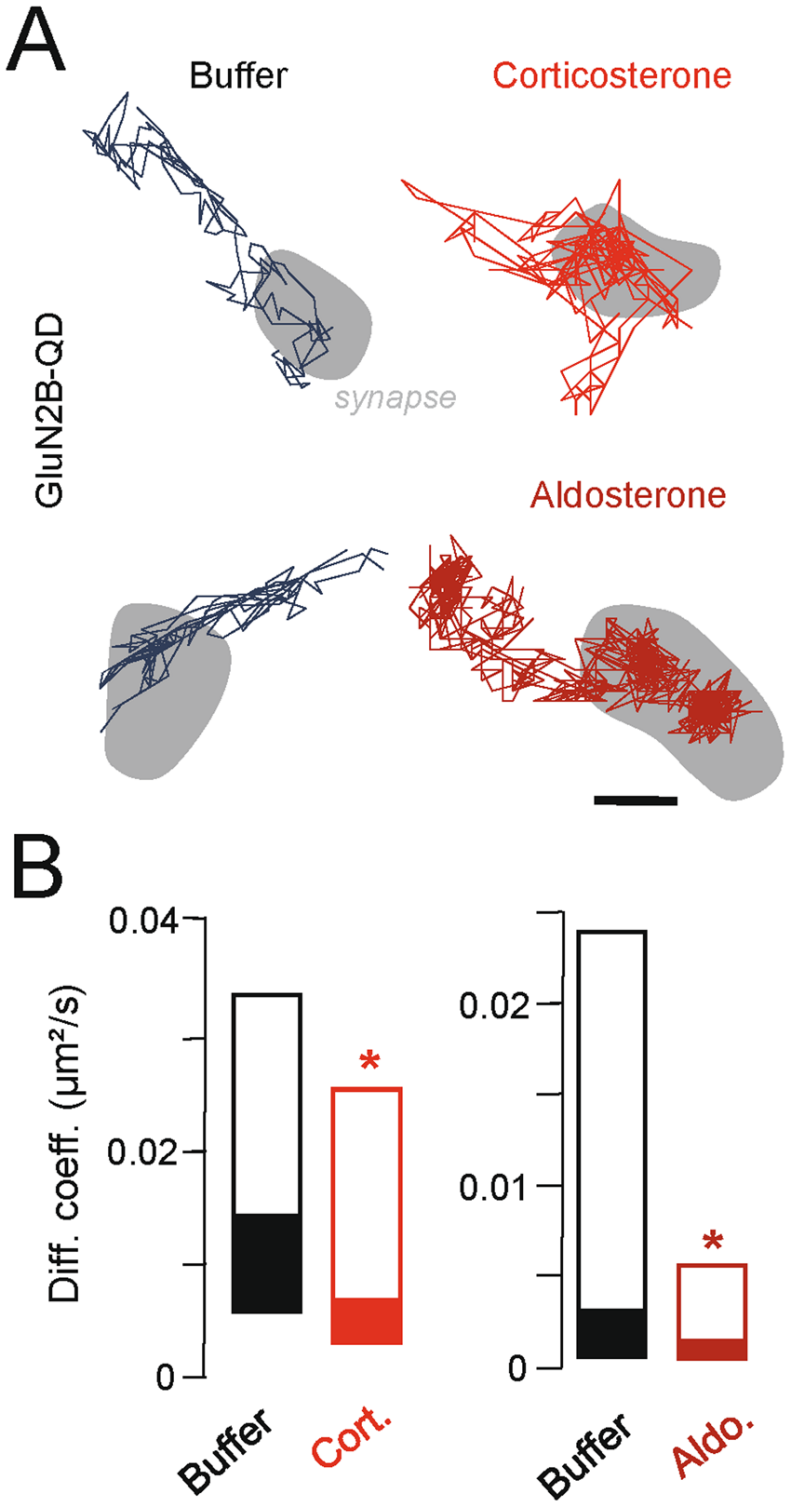
Corticosterone and aldosterone decrease GluN2B-NMDAR surface dynamics within synapses. (**A**) Representative trajectories of surface GluN2B-NMDAR in hippocampal synapses exposed to either buffer solution, aldosterone (10 nM, 20 min), or corticosterone (100 nM, 20 min). Scale bar, 200 nm. (**B**) Comparison of the synaptic GluN2B-NMDAR instantaneous diffusion coefficients between buffer and corticosterone (buffer, n = 141 trajectories; corticosterone, n = 138 trajectories), and buffer and aldosterone (buffer, n = 291 trajectories; aldosterone, n = 402 trajectories). Data are expressed as median diffusion coefficient ±25–75% IQR. *p < 0.05, two-tailed Mann-Whitney test.

Finally, the effect of corticosterone on the GluN2B-NMDAR synaptic content could originate from a higher retention of the receptors within the synapse. Indeed, we previously showed that changes in the synaptic surface dynamics of surface GluN2-NMDAR alter their synaptic content^15, 33–35^. Few minutes after corticosterone exposure, GluN2B-NMDAR synaptic diffusion was significantly reduced, consistent with a higher anchoring of the receptors in the synaptic area (Fig. 6A,B). This effect could be mimicked by the application of aldosterone (10 nM), consistent with the implication of a MR-like signalling in this synaptic retention of GluN2B-NMDAR (Fig. 6A). Noteworthy, the effect of corticosterone and aldosterone on synaptic GluN2B-NMDAR was higher than the ones observed for extrasynaptic GluN2B-NMDAR (e.g. corticosterone effect on synaptic receptor, −95%, and extrasynaptic receptor, −64%) synaptic effect of corticosterone (Figs 4, 5 and 6). Although the mechanism is still undefined, the stabilization of GluN2B-NMDAR by MR-related kinase signalling is likely^36^. Together, these data unravel that corticosterone rapidly activates MR, leading to an efficient postsynaptic anchoring of GluN2B-NMDAR.

### Corticosterone-induced AMPAR synaptic potentiation requires NMDAR surface redistribution

**Figure 7:**
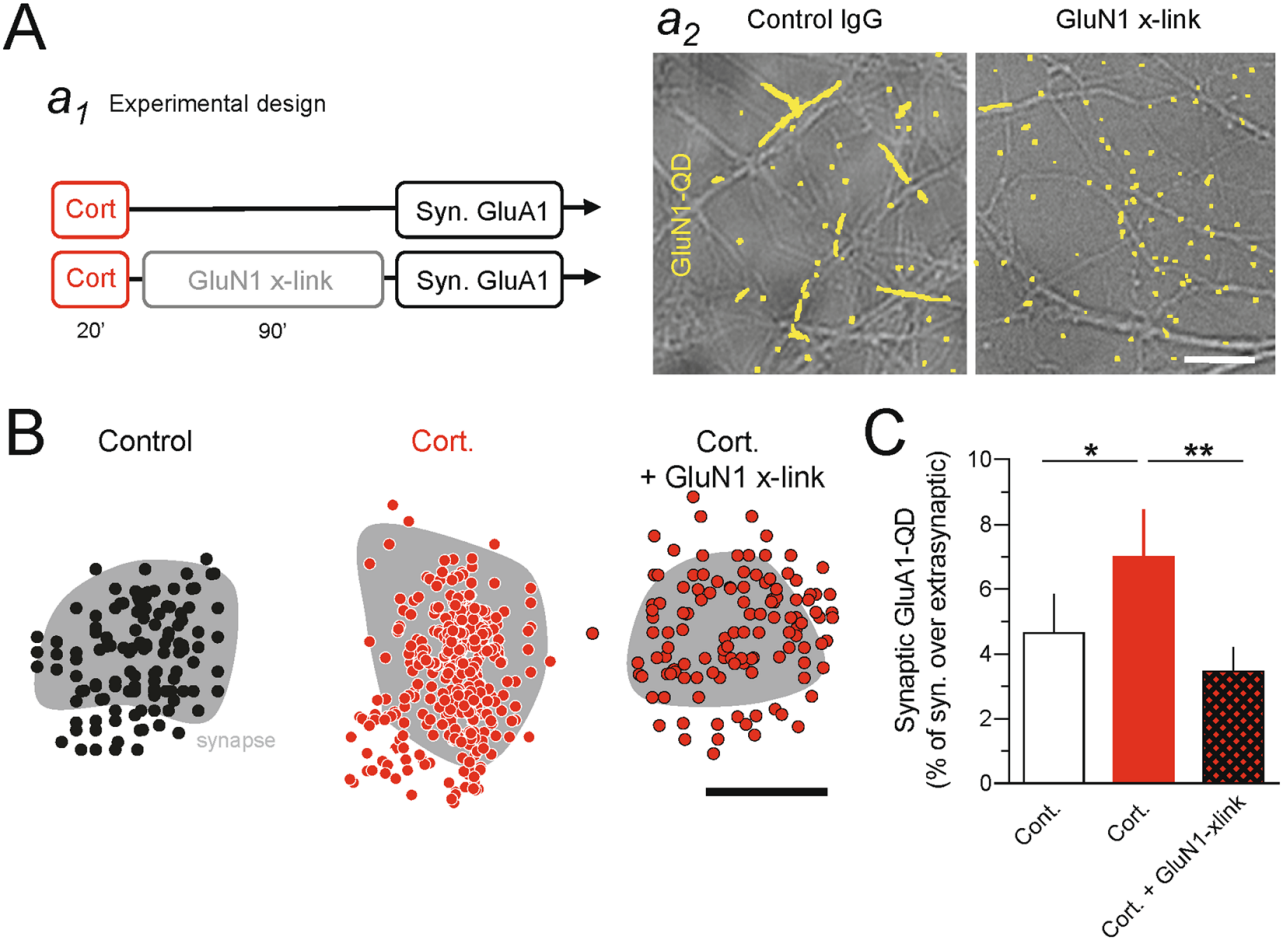
Corticosterone-induced AMPAR synaptic increase is prevented by surface NMDAR cross-linking (x-link). (**A**) Schematic representation of the experimental design (*a*_1_). Characteristic effect of GluN1 cross-linking on GluN1-NMDAR surface diffusion. Note the strong reduction in trajectory lengths in GluN1 x-link (20 min exposure) when compared to the control IgG condition (*a*_2_). Scale bar, 4 µm. (**B**) Representative distributions of GluA1-AMPAR within synapses exposed to corticosterone alone or corticosterone plus GluN1 x-link. Scale bar, 500 nm. (**C**) Comparison of the percent of synaptic GluA1-AMPAR over the extrasynaptic ones between conditions (control, n = 26 dendritic fields; corticoterone, n = 28 dendritic fields; corticosterone + GluN1 x-link, n = 32 dendritic fields; N > 7 neurons for each condition). *p < 0.05, **p < 0.01, One-way ANOVA followed by a Newman-Keuls multiple comparison test.

The fast remodeling of synaptic GluN2B-NMDAR triggered by corticosterone might theoretically impact the synaptic long-term adaptations observed after stress hormone exposure. Among these, it is well-documented that corticosterone induces long-term potentiation (LTP) of AMPAR in hippocampal synapses, which occlude classical NMDAR-dependent theta-burst and tetanus-induced LTP^37^. At the cellular level, this effect is mediated by an increased trafficking of AMPAR toward and within the plasma membrane^25, 26^. Since corticosterone rapidly primes glutamate synapse for potentiation^38^ and since a decrease in the GluN2A/GluN2B ratio favors LTP^11^, we directly tested the intriguing possibility that the surface redistribution of GluN2B-NMDAR observed after corticosterone play an instrumental role in triggering corticosterone-induced AMPAR synaptic potentiation. For this, live hippocampal neurons were incubated with corticosterone for 20 min and the synaptic retention of single GluA1-AMPAR (GluA1-QD) was estimated 90 min later in conditions in which NMDAR were either free to diffuse or immobilize by x-linking^34^ (Fig. 7A). Consistent with previous report^38^, corticosterone increased the relative content of synaptic GluA1-AMPAR when measured 1.5 h after exposure (Fig. 7A,B). Strikingly, reducing the surface diffusion of NMDAR by x-link fully prevented the corticosterone-induced AMPAR synaptic potentiation (Fig. 7B,C). Together, these data provide thus the first evidence that the well-documented effect of corticosterone on synaptic potentiation requires, among the first steps, a rapid redistribution of surface NMDAR.

## Discussion

The plasticity of glutamatergic synapses is altered following acute and chronic stressor exposure, favouring neuronal network and behavioural adaptations^4, 37, 39^. Although changes in NMDAR signalling has long been known to play a key role in synaptic adaptation^11^, the cellular pathway ensuring stress hormone-related and NMDAR-dependent synaptic adaptations has only recently been under scrutiny^18–21^. Using single molecule imaging in rat hippocampal networks, we here uncover that corticosterone regulates NMDAR signalling through a rapid tuning of GluN2B-NMDAR surface dynamics and synaptic content. The GluN2B-NMDAR dynamic changes were triggered by a membrane MR-like pathway and involved an increase stabilization of GluN2B-NMDAR within the glutamatergic synapse area. Functionally, this membrane process directly contributes to the induction of AMPAR synaptic potentiation following exposure to corticosterone, a cellular substrate of the stress-related memory functions^4^. Thus, we unveiled the pathway by which glutamate synapses are rapidly primed for plasticity by corticosterone.

The long-term effect of stress on the cellular expression of NMDAR in the hippocampus and cortex has been well-documented in rodent models. For instance, in the hippocampus and cortex, stress reduces the expression of the main GluN subunits, i.e. GluN1, 2A, and 2B subtypes. Chronic stress decreased, both in juvenile and adult rodents, the protein expression of the GluN2B subunit in the hippocampus^40^. Furthermore, 5 consecutive days of stress exposure are enough to decrease the total and surface amount of GluN1, 2 A, and 2B subunits, via a GR-dependent process^21^. Since chronically-stressed animals exhibit either higher GluN2A mRNA level with no change in GluN2B mRNA^41^ or no change in mRNA levels of GluN1, 2A, and 2B subunit in the hippocampus^42^, transcriptional and/or transductional regulatory mechanisms likely operate under these conditions. Early in life, stress experience also shapes adult neuronal networks and behavioural repertoire^43^. During the postnatal period, a low maternal care increases stress level and decreases the expression of GluN1, 2A, and 2B subunits at the adult stage^44^. Likewise, a prenatal exposure to corticosterone reduces GluN1 subunit mRNA level in the hippocampus^45^. Chronic stress at different developmental stages alters the NMDAR signalling by regulating the receptor subunit expression. A single stressor event or short corticosterone exposure is remarkably sufficient to increase NMDAR EPSCs^18, 20^, and GluN1, GluN2A, and GluN2B subunit expressions^19–21^ in hippocampal and prefrontal cortex neurons. Our macroscopic observations support these observations and further demonstrate that at the surface of hippocampal neurons the synaptic content of GluN2A and GluN2B-NMDAR is rapidly altered by corticosterone. Thus, short and long exposures to corticosterone differentially tune NMDAR signalling in hippocampal networks by altering the expression and synaptic presence of NMDAR subunits, allowing salient adaptations of glutamate synapses.

Understanding the cellular and molecular pathway underlying this physiological process is a key challenge since chronic stress favours the emergence of disabling neuropsychiatric disorders. In cortical neurons, chronic stress decreases NMDAR subunit expression through ubiquitin/proteasome-mediated degradation process, requiring the E3 ubiquitin ligase Fbx2^21^. Acutely, it has been proposed that corticosterone increases the surface content of GluN1, 2A and 2B subunits (without change in the overall subunit content) through the induction of serum- and glucocorticoid-inducible kinase and Rab4 protein. We here shed the first light of the behaviour of membrane NMDAR directly after exposure to corticosterone. Remarkably, corticosterone rapidly tunes NMDAR surface dynamics and synaptic anchoring. This latter process is specific to GluN2B-NMDAR since GluN2A-NMDAR surface dynamics was insensitive to corticosterone, at least in this short time window. Furthermore, impermeable corticosterone and MR agonist resume these effects on GluN2B-NMDAR surface dynamics, supporting a model in which corticosterone activates membrane MR signalling, in a fast and non-genomic manner, to control the membrane diffusion and synaptic anchoring of GluN2B-NMDAR. As GluN2B-NMDAR are among the most diffusive NMDAR at the plasma membrane of hippocampal neurons^15, 33^, the recruitment of such a cellular pathway by corticosterone ensures an efficient, fast, and low cost energy mean to tune network plasticity.

A brief pulse of corticosterone increases the surface expression and synaptic localization of AMPAR and increases the amplitude of miniature excitatory postsynaptic currents^7, 25^. Furthermore, a single exposure to corticosterone occludes the induction of both chemically and electrically evoked LTP^8, 37^, supporting the view that corticosterone- and LTP-dependent processes fully, or partly, overlap^4^. Noteworthy, two corticosterone pulses (60 minutes apart) differentially impact LTP extent and AMPAR trafficking, as we demonstrated that a second corticosterone pulse, mimicking ultradian pulses, restore LTP through a non-genomic glucocorticoid receptor-dependent process^39, 46^. The synaptic ratio between GluN2A and 2B subunits differentially influences the plasticity of glutamate synapses^11^. In addition to long-term changes, rapid modifications of this ratio have been reported shortly after the induction of synaptic plasticity^13, 14^, providing a metaplastic way to encode activity history within synapses. The cellular pathway underlying this process has recently been uncovered, involving a fast redistribution of NMDAR through surface trafficking^34^. Here, we provide the first evidence that corticosterone-induced AMPAR potentiation in hippocampal synapses require a fast redistribution of GluN2B-NMDAR, leading to change of the GluN2A/2B synaptic ratio. Since GluN2B-NMDAR surface dynamics is also required to induce hippocampal LTP *in vitro*, *ex vivo*, and *in viv*o^34, 47^, we thus propose that GluN2B-NMDAR dynamic redistribution is a core mechanism for synaptic plasticity in artificial and physiological conditions. These findings are relevant to the former model proposing that shortly after stress, or stress hormone exposure, NMDAR-dependent LTP is favoured and implicated into flashbulb memory for instance^48^. Our new findings support a model in which the fast molecular dynamics of NMDAR after exposure to stress hormone open a specific plastic window, favouring LTP and memory encoding. Over time, the high surface trafficking returns to basal value, restricting the plastic window and contributing to the memory consolidation. Although the molecular cascade involved in the facilitation of LTP is still unclear, the seminal observation that hippocampal synapses exposed to glucocorticoids exhibit first a NMDA receptor and PKA-dependent insertion of Ca2+-permeable AMPAR, followed by an additional NMDAR-independent form of LTP during high frequency stimulation, support then a multi-stage model: i) corticosterone rapidly “primes” synapses through NMDAR activation, protein kinases recruitment and fast change in GluN2-NMDAR surface distribution, ii) this NMDAR activation subsequently mobilize AMPAR pools to the stimulated synapses, and iii) the “plastic” state of the synapses is thus profoundly affected, likely favouring specific alterations of the neuronal network.

Altogether, this study offers the first evidence, at the single membrane receptor level, that corticosterone controls NMDAR synaptic signalling and synaptic LTP by efficiently tuning GluN2B-NMDAR surface dynamics and anchoring within glutamatergic synapses. Identifying the molecules involve in this unsuspected pathway will thus be of great importance to fully understand the behavioural adaptations triggered by corticosterone. In addition, investigating whether the corticosterone-induced change in NMDAR signalling depend on gender, as demonstrated for other cellular aspects of stress responses^49, 50^, and brain regions^51^ will be of great interest. Finally, it also opens avenues for conceptually new and innovative therapeutical strategies to treat stress hormone-related disorders by focusing, for instance, on the NMDAR trafficking rather than on glutamatergic currents as currently done in most pharmaceutical efforts.

## Methods

All experiments were carried out in accordance with University of Bordeaux and Amsterdam guidelines and regulations.

### Cell culture, immunocytochemistry, synaptic live staining, and protein expression

Cultures of hippocampal neurons were prepared from E18 Sprague-Dawley rats (male and female). The animal procedures were approved by the ethical committee of the University of Bordeaux. Cells, from both dorsal and ventral hippocampi, were plated at a density of 50 × 10^3^ cells per ml on poly-lysine pre-coated cover slips. Coverslips were maintained in a 3% horse serum containing neurobasal medium. This medium is replaced after 4 days *in vitro* (DIV) by a serum-free neurobasal medium. Cultures were maintained at 37 °C in 5% CO_2_ for 20 div at maximum. For surface NMDAR immunostaining, GluN2A-Flag and GluN2B-SEP subunits were co-transfected in neurons at 8–10 div. To image glutamatergic synapses, Homer 1c-DsRed was also co-transfected. Surface GluN2A-Flag and GluN2B-SEP subunits were stained in live neurons using antibodies against FLAG (Sigma, mouse, 1/500, 15 min, 37 °C) and SEP/GFP (R & D systems, rabbit, 1/500, 15 min, 37 °C), respectively. Neurons were then fixed (paraformaldehyde 4%) and incubated with secondary antibody directed against anti-rabbit Alexa 635 antibodies (Invitrogen, 1/500, 45 min) and anti-mouse Alexa 488 antibodies (Invitrogen, 1/500, 45 min). Neurons were washed, mounted, and preparations were kept at 4 °C until observation.

### Time-lapse imaging

Neurons co-transfected with Homer1c-DsRed and either GluN2A-SEP or GluN2B-SEP were placed on the heated stage (37 °C) of an inverted confocal spinning-disk microscope (Leica). To test the population of surface GluN subunits-SEP, we used low pH-solution adjusted to pH 5.4 which quenched all the fluorescence indicating that SEP allows the specific visualization of surface receptors^33^. Fluorescence was excited using a monochromator and cluster fluorescence intensity was followed over time to assess synaptic receptor content. Clusters were imaged over a total period of 35 minutes (corticosterone was applied after a 5 min baseline; the medium was carefully replaced by new equilibrated and heated medium after the protocol application). Fluorescence intensity was measured using Metamorph software (Universal imaging, USA) and corrected for photobleaching and background noise.

### Single Quantum Dot tracking

Single particle detection and imaging were performed as previously described in details (24, 25). Schematically, nanoparticle Quantum dots (QD) 655 Goat F(ab’)2 anti-Rabbit IgG (Invitrogen, CA, USA) were first incubated for 30 min with 1 µl of monoclonal antibodies directed against GluN1, GluN2A, GluN2B and GluA1 subunit (Origin). Non-specific binding was blocked by additional casein (Vector Laboratories, USA) to the QD 15 min before use. Neurons were then incubated for 10 min with pre-coated anti-GluN1, GluN2A, GluN2B and GluA1 subunit QD (final dilution 1:10000) and mounted on a heated-chamber for observation. QD were detected by using a mercury lamp, an oil immersion objective (x100 Apo, N.A. 1.45, W.D. 0.13 mm), and appropriate excitation/emission filters (excitation 435/40–25, emission 655/15–25, dichroic 510 nm; Semrock, Rochester, US) on a wide-field epifluorescent microscope (Nikon, Japan). Images were obtained with an acquisition time of 50 ms with up to 1–1000 consecutive frames. Signals were detected using an EM-CCD camera (Evolve, Roper Scientific) and a pointing accuracy of ~20 nm (pixel size at the imaging magnification = 160 nm). QD were followed on randomly selected dendritic regions and imaging session on a labelled neuronal preparation last to a maximum of 20 min. QD recording sessions were processed with the Metamorph software (Universal Imaging Corp). All recording sessions were acquired within 30 min following primary antibody incubation to minimize receptor endocytosis. The instantaneous diffusion coefficient ‘D’ was calculated for each trajectory, from linear fits of the first 4 points of the mean-square-displacement versus time function using MSD(t) =  <r^2^> (t) = 4Dt. The two-dimensional trajectories of single molecules in the plane of focus were constructed by correlation analysis between consecutive images using a Vogel algorithm. To determine the distribution and synaptic fraction of single QD complexes, 500–1000 frame stacks were performed while tracking down a single QD complex in synapses that were labelled with Mitotracker^52^. The precise location of the receptor/particle complex was determined on each frame and those 500–1000 locations were then projected on a single image, providing a high-resolution map of the successive positions of the receptor/particle complex during the stack. Synaptic and extrasynaptic receptor/particle complex locations were then defined for all receptors of a given neuronal field with respect to Mitotracker labelling. The effects of the different drugs were tested in separate neuronal cultures.

### Drug applications

The following drugs were directly applied in culture medium: corticosterone (water soluble 2-hydroxypropyl-β-cyclodextrin complex) (Sigma-Aldrich; 50–100 nM; dissolved in PBS); corticosterone-BSA (bovine serum albumin) (Steraloids Inc, UK; 50 nM; dissolved in PBS); aldosterone, a selective MR agonist (Sigma-Aldrich; 10 nM; dissolved in 0.0003% ethanol (EtOH)); RU28362, a selective GR agonist (Sigma-Aldrich; 50 nM; dissolved in 0.005% EtOH). In most cases (otherwise stated), drugs were bath applied for 20 min before investigating their effects. As previously described for the x-link experiments^34^, neurons were incubated with highly concentrated (1:10) polyclonal antibodies directed against GluN1 (Alomone Labs; epitope corresponding to residues 385–399 of the GluN1 subunit).

### Electrophysiology

Coverslips were placed in a recording chamber mounted on an upright microscope (Zeiss Axioskop 2 FS Plus, Germany). We used Mg^2+^ free extracellular solution which contained the following component (in mM); 140 NaCl, 5 KCl, 3 CaCl2, 10 Glucose, 10 HEPES, 0.0025 TTX, 0.02 bicuculline, 0.005 NBQX, pH 7.4 (310 mOsm), and kept the coverslip fully submerged. Corticosterone (100 nM, Sigma) or vehicle solution (<0.1% ethanol) was added directly into the extracellular solution while recording. In a separate series of experiments, testing the involvement of GluN2B subunits, the selective GluN2B- blocker Ro 25–6981 (3 μM, Tocris) or vehicle (DMSO) was also directly added into the extracellular solution while recording. Whole cell patch clamp recordings were made using an AXOPATCH 200B amplifier (Axon Instruments, USA), with electrodes from borosilicate glass (1.5 mm outer diameter, Hilgerberg, Malsfeld, Germany). The electrodes were pulled on a Sutter (USA) micropipette puller. The pipette solution contained (in mM): 120 Cs methane sulfonate; CsCl (17.5); HEPES (10); BAPTA (5); Mg-ATP (2); Na-GTP (0.5); QX-314 (10); pH 7.4, adjusted with CsOH; pipette resistance was between 3–6 MΩ. Under visual control (40X objective and 10X ocular magnification) the electrode was directed towards a neuron with positive pressure. Once sealed on the cell membrane (resistance above 1 GΩ) the membrane patch under the electrode was ruptured by gentle suction and the cell was kept at a holding potential of −70 mV. The liquid junction potential caused a shift of no more than 10 mV, which was not compensated during mEPSCs recording. Recordings with an uncompensated series resistance of <15 MΩ and <2.5 times of the pipette resistance and with a shift of <20% during the recording were accepted for analysis. Data acquisition was performed with PClamp 8.2 and analyzed off-line with MiniAnalysis. NMDAR mEPSCs were recorded for 3 minutes. Between the experimental groups (on separate cells) we compared NMDAR mEPSCs after treatment with the drugs (using unpaired t-test). Threshold for detecting NMDAR mEPSCs was 5 pA.

### Data analysis

Because data of diffusion coefficients are not normally distributed, comparisons between groups for instantaneous diffusion coefficients were performed using Mann Whitney test (pair comparison) or Kruskal-Wallis followed by Dunn’s Multiple Comparison Test (group comparison). All the other comparisons between groups for live cell imaging or single cell electrophysiology were performed using parametric statistical tests, Student-t test (unpaired or paired comparison, when appropriate), ANOVA followed by Newman-Keuls Multiple Comparison Test (group comparison), or Kolmogorov-Smirnov test (distribution comparison). Significance levels were defined as *p < 0.05, **p < 0.01, ***p < 0.001.

## Electronic supplementary material

Suppl Fig 1

## References

[1] de Kloet ER, Joels M, Holsboer F (2005). Stress and the brain: from adaptation to disease. Nat Rev Neurosci.

[2] McEwen BS (2007). Physiology and neurobiology of stress and adaptation: central role of the brain. Physiol Rev.

[3] Joels M (2006). Corticosteroid effects in the brain: U-shape it. Trends Pharmacol Sci.

[4] Krugers HJ, Hoogenraad CC, Groc L (2010). Stress hormones and AMPA receptor trafficking in synaptic plasticity and memory. Nat Rev Neurosci.

[5] Karst H (2005). Mineralocorticoid receptors are indispensable for nongenomic modulation of hippocampal glutamate transmission by corticosterone. Proc Natl Acad Sci USA.

[6] Wiegert O, Joels M, Krugers H (2006). Timing is essential for rapid effects of corticosterone on synaptic potentiation in the mouse hippocampus. Learn Mem.

[7] Karst H, Joels M (2005). Corticosterone slowly enhances miniature excitatory postsynaptic current amplitude in mice CA1 hippocampal cells. J Neurophysiol.

[8] Kim JJ, Diamond DM (2002). The stressed hippocampus, synaptic plasticity and lost memories. Nat Rev Neurosci.

[9] Collingridge GL, Isaac JT, Wang YT (2004). Receptor trafficking and synaptic plasticity. Nat Rev Neurosci.

[10] Cull-Candy S, Brickley S, Farrant M (2001). NMDA receptor subunits: diversity, development and disease. Curr Opin Neurobiol.

[11] Yashiro K, Philpot BD (2008). Regulation of NMDA receptor subunit expression and its implications for LTD, LTP, and metaplasticity. Neuropharmacology.

[12] Paoletti P, Bellone C, Zhou Q (2013). NMDA receptor subunit diversity: impact on receptor properties, synaptic plasticity and disease. Nat Rev Neurosci.

[13] Bellone C, Nicoll RA (2007). Rapid bidirectional switching of synaptic NMDA receptors. Neuron.

[14] Matta JA, Ashby MC, Sanz-Clemente A, Roche KW, Isaac JT (2011). mGluR5 and NMDA receptors drive the experience- and activity-dependent NMDA receptor NR2B to NR2A subunit switch. Neuron.

[15] Groc L (2006). NMDA receptor surface mobility depends on NR2A-2B subunits. Proc Natl Acad Sci USA.

[16] Tovar KR, Westbrook GL (2002). Mobile NMDA receptors at hippocampal synapses. Neuron.

[17] Zhao J (2008). Synaptic metaplasticity through NMDA receptor lateral diffusion. J Neurosci.

[18] Tse YC, Bagot RC, Hutter JA, Wong AS, Wong TP (2011). Modulation of synaptic plasticity by stress hormone associates with plastic alteration of synaptic NMDA receptor in the adult hippocampus. PLoS One.

[19] Yuen EY (2009). Acute stress enhances glutamatergic transmission in prefrontal cortex and facilitates working memory. Proc Natl Acad Sci USA.

[20] Yuen EY (2011). Mechanisms for acute stress-induced enhancement of glutamatergic transmission and working memory. Mol Psychiatry.

[21] Yuen EY (2012). Repeated stress causes cognitive impairment by suppressing glutamate receptor expression and function in prefrontal cortex. Neuron.

[22] Rathje M (2013). AMPA receptor pHluorin-GluA2 reports NMDA receptor-induced intracellular acidification in hippocampal neurons. Proc Natl Acad Sci USA.

[23] Ashby MC, Maier SR, Nishimune A, Henley JM (2006). Lateral diffusion drives constitutive exchange of AMPA receptors at dendritic spines and is regulated by spine morphology. J Neurosci.

[24] Mikasova L (2012). Disrupted surface cross-talk between NMDA and Ephrin-B2 receptors in anti-NMDA encephalitis. Brain.

[25] Groc L, Choquet D, Chaouloff F (2008). The stress hormone corticosterone conditions AMPAR surface trafficking and synaptic potentiation. Nat Neurosci.

[26] Martin S (2009). Corticosterone alters AMPAR mobility and facilitates bidirectional synaptic plasticity. PLoS ONE.

[27] Lau CG, Zukin RS (2007). NMDA receptor trafficking in synaptic plasticity and neuropsychiatric disorders. Nat Rev Neurosci.

[28] Groc L (2004). Differential activity-dependent regulation of the lateral mobilities of AMPA and NMDA receptors. Nat Neurosci.

[29] Michaluk P (2009). Matrix metalloproteinase-9 controls NMDA receptor surface diffusion through integrin beta1 signaling. J Neurosci.

[30] Groc L, Choquet D (2008). Measurement and characteristics of neurotransmitter receptor surface trafficking (Review). Mol Membr Biol.

[31] Groc L (2007). Surface trafficking of neurotransmitter receptor: comparison between single-molecule/quantum dot strategies. J Neurosci.

[32] Bard L, Groc L (2011). Glutamate receptor dynamics and protein interaction: Lessons from the NMDA receptor. Mol Cell Neurosci.

[33] Bard L (2010). Dynamic and specific interaction between synaptic NR2-NMDA receptor and PDZ proteins. Proc Natl Acad Sci USA.

[34] Dupuis JP (2014). Surface dynamics of GluN2B-NMDA receptors controls plasticity of maturing glutamate synapses. EMBO J.

[35] Groc L (2007). NMDA receptor surface trafficking and synaptic subunit composition are developmentally regulated by the extracellular matrix protein Reelin. J Neurosci.

[36] Yang S, Roselli F, Patchev AV, Yu S, Almeida OF (2013). Non-receptor-tyrosine kinases integrate fast glucocorticoid signaling in hippocampal neurons. J Biol Chem.

[37] Chaouloff F, Groc L (2011). Temporal modulation of hippocampal excitatory transmission by corticosteroids and stress. Front Neuroendocrinol.

[38] Joels M, Baram TZ (2009). The neuro-symphony of stress. Nat Rev Neurosci.

[39] Sarabdjitsingh RA (2014). Ultradian corticosterone pulses balance glutamatergic transmission and synaptic plasticity. Proc Natl Acad Sci USA.

[40] Li SX (2014). Role of the NMDA receptor in cognitive deficits, anxiety and depressive-like behavior in juvenile and adult mice after neonatal dexamethasone exposure. Neurobiol Dis.

[41] Costa-Nunes J (2014). Altered emotionality, hippocampus-dependent performance and expression of NMDA receptor subunit mRNAs in chronically stressed mice. Stress.

[42] Suenaga T, Morinobu S, Kawano K, Sawada T, Yamawaki S (2004). Influence of immobilization stress on the levels of CaMKII and phospho-CaMKII in the rat hippocampus. Int J Neuropsychopharmacol.

[43] Krugers, H. J. & Joels, M. Long-lasting Consequences of Early Life Stress on Brain Structure, Emotion and Cognition. *Curr Top Behav Neurosci* (2014). 10.1007/7854_2014_289 24862989

[44] Bagot RC (2012). Maternal care influences hippocampal N-methyl-D-aspartate receptor function and dynamic regulation by corticosterone in adulthood. Biol Psychiatry.

[45] Owen D, Matthews SG (2007). Repeated maternal glucocorticoid treatment affects activity and hippocampal NMDA receptor expression in juvenile guinea pigs. J Physiol.

[46] Sarabdjitsingh RA (2016). Hippocampal Fast Glutamatergic Transmission Is Transiently Regulated by Corticosterone Pulsatility. PLoS One.

[47] Potier, M. *et al*. Temporal Memory and Its Enhancement by Estradiol Requires Surface Dynamics of Hippocampal CA1 N-Methyl-D-Aspartate Receptors. *Biol Psychiatry* (2015). 10.1016/j.biopsych.2015.07.017 26321020

[48] Diamond DM, Campbell AM, Park CR, Halonen J, Zoladz PR (2007). The temporal dynamics model of emotional memory processing: a synthesis on the neurobiological basis of stress-induced amnesia, flashbulb and traumatic memories, and the Yerkes-Dodson law. Neural Plast.

[49] Palomero-Gallagher N, Bidmon HJ, Zilles K (2003). AMPA, kainate, and NMDA receptor densities in the hippocampus of untreated male rats and females in estrus and diestrus. J Comp Neurol.

[50] Kitraki E, Kremmyda O, Youlatos D, Alexis MN, Kittas C (2004). Gender-dependent alterations in corticosteroid receptor status and spatial performance following 21 days of restraint stress. Neuroscience.

[51] Grigoryan G, Ardi Z, Albrecht A, Richter-Levin G, Segal M (2015). Juvenile stress alters LTP in ventral hippocampal slices: involvement of noradrenergic mechanisms. Behav Brain Res.

[52] Ehlers MD, Heine M, Groc L, Lee MC, Choquet D (2007). Diffusional trapping of GluR1 AMPA receptors by input-specific synaptic activity. Neuron.

