# Supplementary figures and images for: Stress hormone rapidly tunes synaptic NMDA receptor through membrane dynamics and mineralocorticoid signalling
Source: Sci Rep. 2017 Aug 14;7:8053. doi: 10.1038/s41598-017-08695-3 (PMC5556050; doi:10.1038/s41598-017-08695-3)

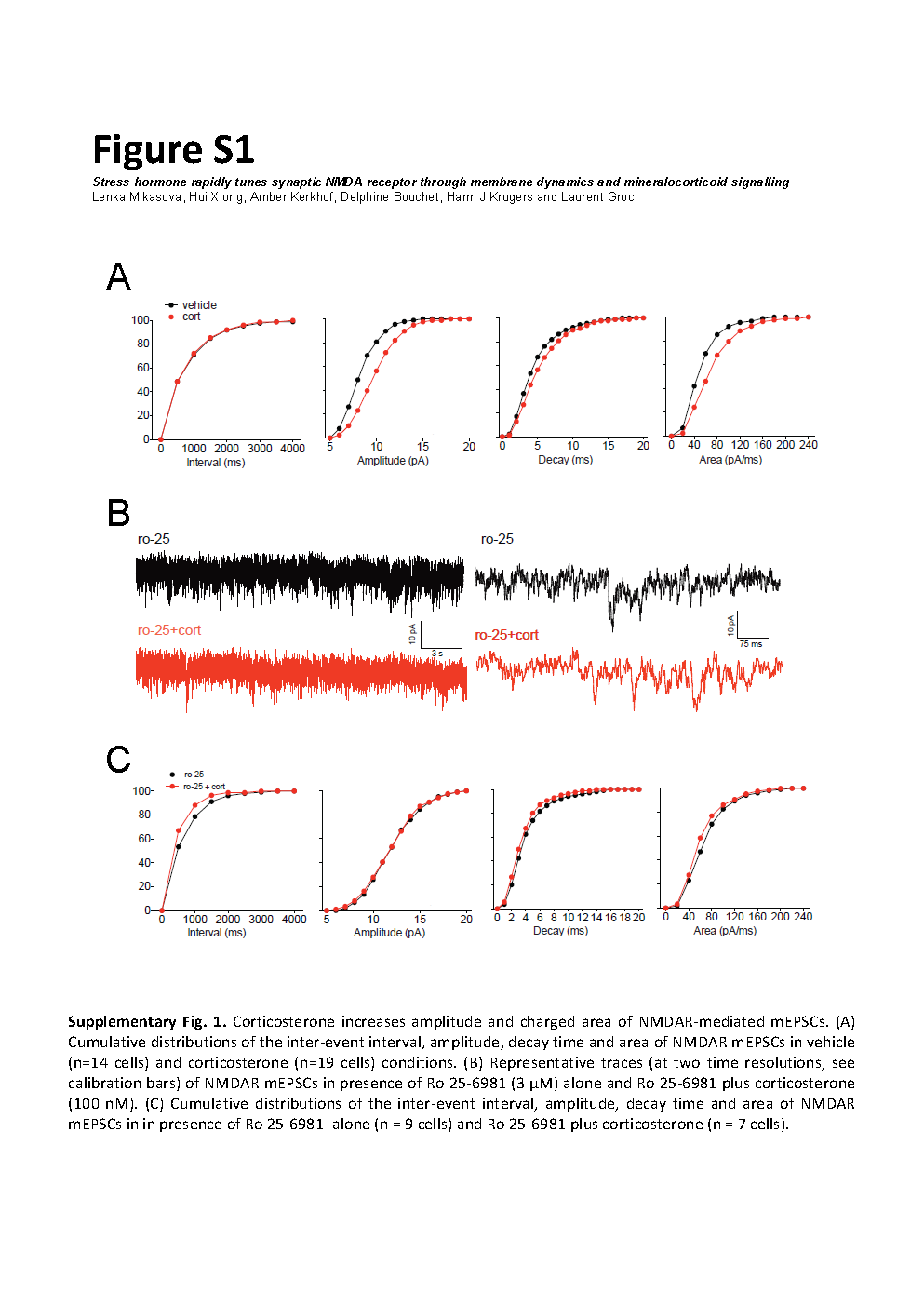

Supplement: Supplementary file 1 — Suppl Fig 1 [file 41598_2017_8695_MOESM1_ESM.tif]
